# Data on occurrence and ecotoxicological risk of emerging contaminants in Dinaric karst catchment of Jadro and Žrnovnica springs

**DOI:** 10.1016/j.dib.2022.108157

**Published:** 2022-04-10

**Authors:** Ana Selak, Jasmina Lukač Reberski, Göran Klobučar, Ivana Grčić

**Affiliations:** a Department of Hydrogeology and Engineering Geology, HGI-CGS – Croatian Geological Survey, Sachsova 2, Zagreb 10000, Croatia; b Department of Biology, Division of Zoology, PMF – Faculty of Science, Rooseveltov trg 6, Zagreb 10000, Croatia; c Department of Environmental Engineering, GFV – Faculty of Geotechnical Engineering, Hallerova aleja 7, Varaždin 42000, Croatia

**Keywords:** Emerging contaminants, Persistence, Bioaccumulation, Toxicity, Mobility, Risk quotient (RQ), Water, Karst

## Abstract

Karst catchments are valuable drinking water sources and fragile habitats to many endemic species. This dataset presents initial insights into the occurrence and ecotoxicological risk of 21 emerging contaminants (ECs) (including 11 pharmaceuticals, 4 lifestyle products, 2 personal care products, 3 agricultural and 1 industrial compound) detected in Dinaric karst catchment of Jadro and Žrnovnica springs in Croatia. Contaminants concentrations were determined with UHD Q-TOF LC/MS and UHP LC/MS in samples from two springs (Jadro and Žrnovnica), one river (Cetina), and a deep borehole (Gizdavac). Persistence (P), bioaccumulation (B), mobility (M) and toxicity (T) of detected ECs were assessed based on *in silico* strategy for PBT assessment and recently developed REACH PMT/vPvM guidelines. Risk quotients were calculated from PNEC values and measured contaminants’ concentrations. In addition, physicochemical properties (estimated and existing experimental values of solubility in water, log K_OW_, log K_OC_, and pK_a_) of detected substances and water (measured values of temperature and electrolytic conductivity) are provided. This dataset could be useful for setting up the regular monitoring and improvement of existing water-related legislative, water safety plans, for modelling contaminant transport and identification of potential sources, and lastly for comparison with other studies conducted in karst aquifers.

The present dataset was interpreted and discussed in the article entitled “*Ecotoxicological aspects related to the occurrence of emerging contaminants in the Dinaric karst aquifer of Jadro and Žrnovnica springs*” by Selak et al. (2022).

## Specifications Table

**Table:** 

Subject	Environmental Science
Specific subject area	Ecotoxicological prioritization of emerging contaminants in water
Type of data	Tables Figure
How the data were acquired	Field monitoring and sampling; laboratory analysis (liquid chromatography-mass spectrometry); existing database research; use of EPI Suite™ software; use of Prometheus software; use of PMT/vPvM guidelines; use of QSAR software; use of ToxTree software; use of risk quotient methodology
Data format	Raw Analyzed
Description of data collection	Data was obtained by analysing surface water and groundwater samples collected at 4 sampling points in the karst catchment of Jadro and Žrnovnica springs. Electrolytic conductivity and water temperature were observed at all sampling sites. PBT values of detected ECs were estimated *in silico*, PMT/vPvM categories were assessed according to REACH guidelines, while their environmental concentrations were used to determine the potential environmental risk that they pose.
Data source location	Institution: Croatian Geological Survey City/Town/Region: Split-Dalmatia County, Jadro and Žrnovnica catchment Country: Croatia Latitude and longitude (and GPS coordinates, if possible) for collected samples/data: Jadro spring 43°32′34.6″N, 16°31′20.6″E; Žrnovnica spring 43°31′24.5″N, 16°34′28.4″E; Cetina River 43°37′02.9″N, 16°43′44.0″E; Gizdavac borehole 43°38′43.7″N, 16°29′07.6″E
Data accessibility	The dataset is hosted on a public repository. Repository name: Mendeley Data Data identification number: 10.17632/byk4tyh4jd.1 Direct URL to data: https://data.mendeley.com/datasets/byk4tyh4jd/1
Related research article	A. Selak, J. Lukač Reberski, G. Klobučar, I. Grčić Ecotoxicological aspects related to the occurrence of emerging contaminants in the Dinaric karst aquifer Sci Total Environ. 825 (2022) 153,827. https://doi.org/10.1016/j.scitotenv.2022.153827

## Value of the Data

• The electrolytic conductivity and water temperature are parameters providing baseline knowledge on groundwater and surface water dynamics and character. They also serve as natural tracers for the determination of the relations between specific locations within the catchment area.
• The measured concentrations and main physicochemical properties of detected emerging contaminants provide initial insights into their occurrence in the karst catchment and allow their ecotoxicological characterisation.
• The persistence, bioaccumulation, mobility, and toxicity, estimated based on quantitative structure-activity relationships QSAR modelling and according to PBT and PMT/vPvM assessment guidelines, enable prioritization of emerging contaminant substances. Calculated PBTr values differentiate locations having no, low, medium, or high cumulative PBT rank.
• Risk quotient (RQ) values represent valuable findings on the potential environmental risks that detected emerging contaminants are posing. RQsite values enable the classification of sites based on cumulative risk quotient and point out sites with the highest potential environmental risk.
• The dataset has a multidisciplinary value, as it can be utilised by hydrogeologists, biologists, chemists, and other researchers dealing with water management or environmental issues, as well as national or regional authorities responsible for monitoring activities or practitioners like water suppliers.
• The dataset can be used as an input for modelling contaminant transport and identification of contamination sources, for prioritizing future (eco)toxicological and hydrochemical research, water safety plans, and lastly for comparison with other studies.

## 1 Data Description

**Fig. 1:**
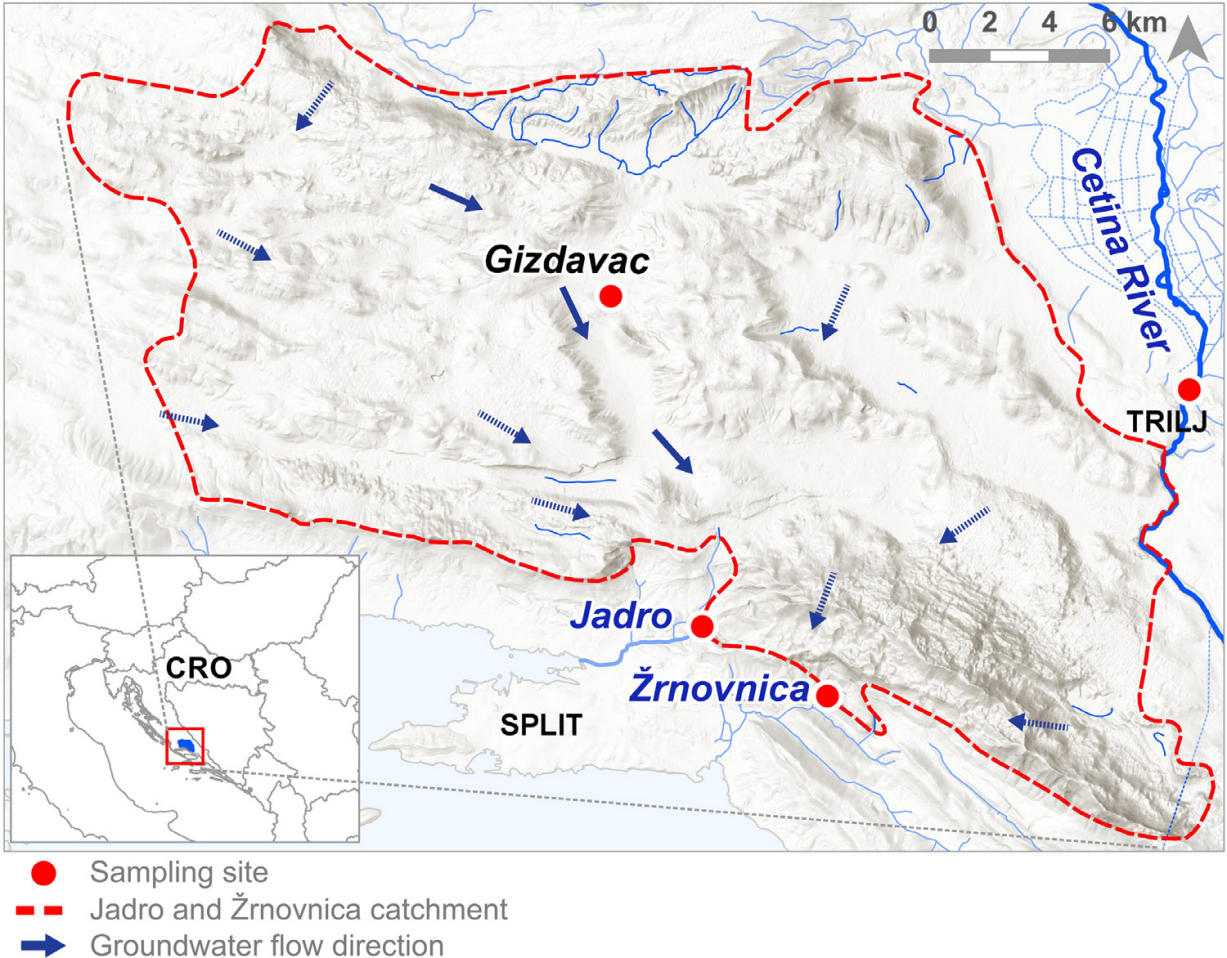
Sampling locations within Jadro and Žrnovnica springs catchment.

The sampling locations chosen within the Dinaric karst catchment of Jadro and Žrnovnica springs (Croatia) are shown in Fig. 1, while their coordinates (in WGS84) are given in Table 1.

**Table 1:** Sampling locations coordinates.

Sampling site	Sampling medium	Latitude (N)	Longitude (E)
Jadro	Spring water	43°32′34.6″	16°31′20.6″
Žrnovnica	Spring water	43°31′24.5″	16°34′28.4″
Cetina	River	43°37′02.4″	16°43′44.6″
Gizdavac	Borehole	43°38′43.7″	16°29′07.6″

The dataset of six tables (Table 2 to 7) in XLSX format is deposited in Mendeley Data online repository [1]. Detection frequency, minimal, maximal, and median concentrations of detected emerging contaminants are shown in Table 2 along with their CAS number, description/use, substance group, limits of detections in two commissioned laboratories (National Laboratory Services, Starcross UK, and Plsen Laboratory Czech Republic), and detection location. Table 3 consists of two worksheets, one showing physicochemical properties of detected emerging contaminants and the other one used references. It includes the molecular formula and weight (g/mol) of each substance, their predicted and existing experimental logarithm of partition coefficient between octanol and water (log K_OW_), organic carbon-water partitioning coefficient (log K_OC_), acid dissociation constant (pK_a_), and contaminants’ solubility in water (mg/L at 25 °C). Physicochemical properties of water (temperature in the first worksheet and electrolytic conductivity in the second worksheet) measured at Jadro and Žrnovnica springs, Cetina River, and Gizdavac borehole, are given in Table 4. Table 5 presents persistence (P), bioaccumulation (B), toxicity (T) values of each contaminant with predicted reliability and scores in the first worksheet. The calculated PBTr values for each sampling site and detected compound are given in the second worksheet of Table 5. In Table 6 PMT/vPvM assessment is given. The first worksheet contains the summary of assessed PMT/vPvM categories. All steps of the PMT/vPvM assessment are given in the second worksheet, while the third one contains used references. Lastly, Table 7 includes the lowest predicted no-effect concentration values (ng/L), measured environmental concentrations (MEC, ng/L), and related risk quotients (RQ) calculated for each site and sampling campaign.

## 2 Experimental Design, Materials and Methods

### 2.1 Measurement of water physicochemical parameters

Physicochemical properties of water (temperature and electrolytic conductivity) were measured using a WTW multi-parameter probe. At Jadro and Žrnovnica springs, measurements were made directly, while at Cetina River parameters were measured in a bucket containing water grabbed from the middle of the river channel. At the Gizdavac borehole, physicochemical parameters were measured in a bucket containing the pumped third volume of groundwater [2].

### 2.2 Analytical methods

The analysis of emerging contaminants in surface and groundwater samples carried out in the course of the GeoTwinn project was done at UK National Laboratory Services at Star Cross with Agilent 6540 Ultra-High-Definition (UHD) Accurate-Mass Quadrupole Time-of-Flight (Q-TOF) liquid chromatography/mass spectrometry (LC/MS) of Agilent Technologies, Inc. (Santa Clara, CA, USA). Sample extraction was done with Waters Oasis HLB SPE cartridges (200 mg) with an automated extraction system. Cartridges were conditioned with 6 mL of methanol followed by 6 mL of Ultra High Purity (UHP) water. The water sample (500 mL, flow rate 10 mL/min) was then loaded onto the cartridge. After loading, the cartridge was washed with 6 mL of UHP water and the sorbent dried fully with high purity nitrogen. The cartridges were then eluted twice, firstly with 6 mL of 0.1% formic acid in methanol:acetonitrile (1:1) and then with 6 mL of dichloromethane (DCM). The elutants are collected in separate vials. The DCM eluate was evaporated to incipient dryness under a gentle stream of nitrogen. Corresponding methanol:acetonitrile eluate is then transferred to the dry DCM vials and evaporated at 100 µL at 35–40 °C. 900 µL of UPH water is added to each of the vials containing the 100 µL extract. The sample is vortexed mixed, filtered, and transferred to a silanized screw-top vial ready for analysis. An isotopically labelled internal standard Carbutamide-d9 was added to each of the pre-conditioned SPE cartridges to assess instrument performance. Target compounds have been analysed using a blank sample and a standard with a concentration of 0.1 μg/L. The response factor obtained is used to create a calibration curve.

Samples collected within the boDEREC—CE project were analysed at the Vltava River Basin Authority laboratory following the EPA method 1694 (Axys Analytical Services, Ltd.) and valid procedures. Upon arrival at the laboratory, the samples were defrosted at a maximum temperature of 30 °C on the day of analysis. The analysis was done with 1290 ultra-high-performance liquid chromatograph (UHPLC in electrospray ionisation ESI+ and ESI- modes) coupled with an Agilent 6495B Triple Quad Mass Spectrometer (MS/MS). Sample preparation included centrifugation in headspace vials for 10 min at about 3500 rpm and weighting of 1.5 g of each sample in a 2 mL vial on the analytical balance. Subsequently, 1.5 μL of acetic acid was added to each sample. An isotope dilution was performed in the next step. Deuterated internal standards of carbamazepine-d_10_, sulfamethoxazole-d_6_, iopromide-d_3_, iopamidol-d_3_, erythromycin-^13^C_2_, ibuprofen-d_3_, diclofenac-d_4_, naproxen-d_3_, chloramphenicol-d_5_ and others were used. The separation was achieved with Waters Xbridge C18 analytical column (100 mm x 4.6 mm i.d., 3.5 μm particle size). Methanol and water with 0.02% acetic acid and 0.5 mM ammonium fluoride were used as the mobile phase additives at the flow rate of 0.5 mL/min. The injection volume was 0.050 mL. Each series of samples was verified by calibration control and by maintaining a clean environment, equipment, and agents. The performance of the analytical system was ensured by blank and spiked samples. The chemicals used for preparing calibration solutions had a certified purity of 99%. Calibration solutions were prepared from neat analytes or solutions with certified concentrations. Each fifth sample in a series was processed by the method of standard addition, which was used to control the effect of the matrix of the sample and to reset the actual recovery ratio of a specific analyte.

### 2.3 Physicochemical properties of emerging contaminants

Physicochemical properties of emerging contaminants were gathered from the publicly available NORMAN Substance Database [3] and PubChem [4]. In the absence of experimental data, those physicochemical properties were estimated using the EPI Suite™ 4.11 interface [5].

### 2.4 PBT ranking

Persistence, bioaccumulation, toxicity (PBT) values of detected emerging contaminants were estimated in Prometheus software [6]. Given their persistence (*P*), toxicity (*T*), and bioaccumulation (*B*) characteristics compounds were scored following Eq. (1), defined by Pizzo et al. [6].

(1) $$\text{PBT}=P^{0.4}\cdot B^{0.4}\cdot T^{0.2}$$

*PBTr* values were obtained based on estimated *PBT* values and measured concentrations of detected emerging contaminants, as proposed by Babić et al. [7] Eq. (2):

(2) $$\text{PBTr}=\frac{\sum_{i=1}^{n}R_{i}w_{i}}{\sum_{i=1}{}^{n}w_{i}}$$

where *Ri* is the rank calculated by multiplying PBT score and measured concentration, and *wi* is the weight. Given how PBT score and concentration are seen as factors equally relevant for the potential risk, all weights are set to 1.

### 2.5 PMT/vPvM assessment

The persistence (P), mobility (M), and toxicity (T) criteria of all detected compounds were evaluated according to REACH guidelines for PMT/vPvM assessment [8].

The criterion for persistency is the degradation half-live of ECs in water or sediment not shorter than 40 days, while very persistent substances (vP) are those having the degradation half-live longer than 180 days. The EC is assessed as potential p/vP substance if there is only screening data (results of inherent/readily biodegradable tests) or QSAR data that indicate potential persistency. A category of potential P/vP++ is given to those ECs for which sufficient weight of evidence indicates that P or vP criterion is met, but it is unclear which. In the absence of experimental data, QSAR Toolbox [9] was used to predict the persistence. To predict the ECs biodegradability, ready biodegradability model (IRFMN 1.0.9.) in VEGA QSAR [10] and BIOWIN models (1,3,4, and 5 models, v4.10) in EPI Suite™ were utilized. The half-lifes were also searched in CompTox Chemicals Dashboard [11].

Experimental log K_OC_ values (within 4–9 pH range) as basis for the mobility criterion were obtained from the literature and PubChem database, or predicted with KOCWIN v2.00 model in EPI Suite™. The substances with log K_OC_ values of ≤4.0 are considered as mobile (M), while those having values of ≤3.0 are very mobile (vM).

The toxicity criterion (T) is fulfilled if EC has either: the long-term no-observed effect concentration (NOEC) or EC10 for freshwater organisms less than 0.01 mg/l; or if it is carcinogenic, germ cell mutagenic, or toxic for reproduction; or if there is other evidence of chronic toxicity such as specific target organ toxicity after repeated exposure. The ECHA Classification and labelling inventory [12] was searched for toxicity data. NOEC values were acquired from EnviroTox database [13]. In case no experimental data was available, models within VEGA QSAR software were used to predict the mutagenicity, carcinogenicity and toxicity. In case none of the above criteria is met, Cramer classification was done in Toxtree v3.1.0.1851 software [14] to differentiate potential toxic substances (pT) having Cramer Class III and non-toxic substances with assessed Cramer Class II or I.

Depending on which criteria are met, six categories are defined [8]

• *vPvM & PMT* (there is sufficient weight of evidence that the EC meets the vP, vM, and T criteria);
• *vPvM* (there is sufficient weight of evidence that the EC meets both the vP and vM criteria, but not the T criterion; this category is given also to substances suspected to be potential P/vP++ if they are detected in raw or drinking water);
• *PMT* (there is sufficient weight of evidence that the EC meets the P, M, and T criteria);
• *PM* (there is sufficient weight of evidence that the ECs meets both the P and M criteria, but does not meet the T criterion nor the vPvM criteria);
• *potential PMT/vPvM* (only screening or low-quality data is available for P, M or both, and that either a conclusion of “potential P/vP” and/or “potential M/vM” is obtained);
• and *not PMT/vPvM* (the “not P” or “not M” criteria was met with sufficient weight of evidence).

### 2.6 Risk quotients

Risk quotient (*RQ*) values were obtained by multiplying measured environmental concentrations of detected emerging contaminants (*MEC*) and the lowest Predicted No-Effect Concentration (*PNEC*) values gathered from the Ecotox database [15]. The lowest *PNEC* value of 1.65 µg/L for ibuprofen was retrieved from the published literature [16]. Four classes of potential ecotoxicological risk of the target compound to the receiving aquatic ecosystems are determined as follows: RQ<0.01 for negligible risk, 0.01<RQ<0.1 for low risk, 0.1<RQ<1 for moderate risk, and RQ>1 for high risk [17,18].

The sum of *RQ* values for all compounds detected at the particular sampling location indicates the site-specific risk quotient *RQ_site_* [19]:

(3) $$RQ_{\text{site}}=\sum_{i=1}^{n}RQ_{i}$$

## 3 Ethics Statements

Not relevant.

## Funding

This work was supported by the Interreg Central Europe boDEREC—CE project co-funded by the 10.13039/501100008530European Regional Development Fund and Horizon 2020 GeoTwinn project under (Grant 809943).

## CRediT authorship contribution statement

**Ana Selak:** Conceptualization, Methodology, Data curation, Writing – original draft, Visualization, Investigation. **Jasmina Lukač Reberski:** Conceptualization, Supervision, Investigation, Writing – review & editing. **Göran Klobučar:** Supervision, Writing – review & editing. **Ivana Grčić:** Supervision.

## Declaration of Competing Interest

The authors declare that they have no known competing financial interests or personal relationships that could have appeared to influence the work reported in this paper.

## Data Availability

Dataset on occurrence and ecotoxicological risk of emerging contaminants in Dinaric karst catchment of Jadro and Žrnovnica springs (Original data) (Mendeley Data).
